# Correlates of suicide risk among Black and White adults with behavioral health disorders in criminal-legal systems

**DOI:** 10.1186/s12888-022-03803-8

**Published:** 2022-03-04

**Authors:** Spencer G. Lawson, Evan M. Lowder, Bradley Ray

**Affiliations:** 1 School of Criminal Justice, Michigan State University, East Lansing, MI USA; 2 Department of Criminology, Law and Society, George Mason University, Fairfax, VA USA; 3 Division for Applied Justice Research, RTI International, Research Triangle Park, NC USA

**Keywords:** Suicide, Correlates, Race, Criminal justice, Behavioral health

## Abstract

**Background:**

Adults with behavioral health disorders in criminal-legal systems are at heightened risk of suicide relative to the general population. Despite documented racial disparities in criminal processing and behavioral health treatment, few studies have examined racial differences in suicide risk in this already high-risk population. This study examined 1) the correlates of suicide risk in this population overall and by race and 2) the moderating role of race in these associations.

**Methods:**

We investigated correlates of clinician-rated suicide risk at baseline in a statewide sample of 2,827 Black and 14,022 White adults with criminal-legal involvement who engaged in community-based behavioral health treatment. Regression-based approaches were used to model suicide risk and test for evidence of interaction effects.

**Results:**

Findings showed the strongest correlates of suicide risk were greater behavioral health needs, evidence of self-harm, and a primary mental health diagnosis or co-occurring diagnosis. In race-specific analyses, correlates of suicide risk were mostly similar for both Black and White clients, with a couple exceptions. Interaction terms testing between-group effects on correlates of suicide risk were non-significant.

**Conclusions:**

Adults with behavioral health disorders in criminal-legal systems experience similar risk factors for suicide as the general population. Similar to prior research, we found that Black adults, in particular, are at lower risk for suicide overall. Contrary to expectations, we found similarities in correlates of suicide risk across race in our sample of felony-level adults with behavioral health disorders in the criminal-legal system. Prior research shows that behavioral health professionals should be cognizant of cultural factors when developing a comprehensive approach to suicide care and treatment. Our findings show correlates of suicide risk are largely stable in Black and White adults involved in criminal-legal systems, suggesting culturally responsive treatment for suicide risk should target shared risk factors.

## Background

Suicide is the 10^th^ leading cause of death in the United States [1], with approximately one death occurring every 12 min [2]. Over the past two decades, deaths by suicide have increased substantially in many states [3], contributing to a 35% growth in the suicide rate from 1999 to 2018 (i.e., 10.5 per 100,000 residents to 14.2) [4]. Suicide attempts and ideation occur with even more frequency with 1.4 million adults having a nonlethal suicide attempt, 3.3 million making a suicide plan, and 10.7 million giving serious thought about killing themselves [5]. Prior research identifies several risk factors for suicide, including access to lethal means, mental health and substance use disorders, prior suicide attempts and ideation, and deliberate self-harm [6, 7].

A half century of scholarship, however, shows that suicide risk is heterogeneously distributed in society, with certain subpopulations at greater risk of suicide relative to others [8, 9]. One group with elevated risk of suicide relative to the general population—and where there are missed opportunities for intervention—are adults who are involved in criminal-legal systems (e.g., law enforcement, jails, courts, prisons, and community supervision) [10, 11]. Research on criminal-legal populations suggests impairment of interpersonal relationships and prior victimization or trauma [12–14] are risk factors for suicide as are experiences unique to criminal-legal involvement; for example, offense type, frequency of system contact, and prior incarcerations [15–18]. Moreover, criminal-legal systems continue to be overrepresented by persons with behavioral health conditions as 60% of individuals incarcerated in prisons and jails met the Diagnostic and Statistical Manual of Mental Disorders 4^th^ edition (DSM-IV) diagnostic criteria for drug use or dependence [19] and 24% to 40% reporting prior mental health and co-occurring conditions [20, 21], which are both established risk factors for suicide [22, 23].

The process of entry into criminal-legal systems is not always based on justice as racial-ethnic minorities continue to be disproportionately policed, arrested, incarcerated, and sentenced [24–27]. In particular, Black individuals who end up being involved in criminal-legal systems encounter more challenges in community functioning [28–30]. Criminal-legal involvement may also contribute to health disparities between Black and White individuals [31, 32]. Evidence on the relationship between access to community-based treatment services and criminal-legal outcomes has been positive [33, 34]; yet, systemic inequities present in behavioral health care access and treatment across racial groups can further compound health disparities [35–37]. In fact, clinical prediction models of suicide may even be harmful to underserved populations [38]. Together, these trends contribute to limited continuity of care for Black individuals, in particular, with behavioral health conditions and opens the door wider for suicide risk.

Criminal-legal systems are not designed or equipped to adequately deal with complex behavioral health conditions. Systematic racism and inadequate health care systems contribute to Black individuals being more likely to have inaccurate diagnoses and improper treatment of behavioral health conditions [39–41]. Recently, several studies examined suicidality among adults with current and prior criminal-legal involvement [10, 12–14, 16, 42–44], but only one study has specifically focused on racial differences in suicidality among felony-level individuals under community corrections supervision [45]. The authors found unique correlates of suicide attempts and ideation that differentiated racial groups (e.g., lack of insurance, substance dependencies). This work represents a significant and valuable addition to the contemporary criminal-legal literature on race-specific correlates of suicide. Yet, the study did not examine key suicide risk factors, including mental health or co-occurring behavioral health conditions, which are widely prevalent among populations in criminal-legal systems.

With research suggesting an increase in suicide risk among Black individuals in the United States [46, 47], it is important to note that studies find risk factors for suicide differ between Black and White populations. Acculturative stressors [48, 49], erosion of social networks [50, 51], and income and educational inequalities [52, 53] are established risk factors in Black individuals. Protective factors include religious and moral beliefs where suicide is viewed as unacceptable [54, 55] and strong familial networks [50, 56].

To advance research on racial differences in suicide, we report on the correlates of clinician-rated suicide risk using a large statewide dataset from a community-based sample of adults with recent criminal-legal involvement who engaged in behavioral health treatment (*N* = 16,849). This dataset is used to understand which factors elevate the risk of suicide and how these differ between Black and White individuals. Thus, our aims were to examine 1) the correlates of clinician-rated suicide risk in this population overall and by race and 2) the moderating role of race on the effect of suicide correlates on clinician-rated suicide risk.

## Methods

### Study Context

Demographically, the state of Indiana had an estimated population of 6,568,645 in 2015. Individuals who identify as White account for the majority of the citizenry, with Black residents making up only 9.2% of the population in Indiana [57]. Indiana’s imprisonment rate was among the highest for Midwestern jurisdictions at year-end 2015 (412 per 100,000 residents), fifth only to South Dakota, Michigan, Ohio, and Missouri [58]. Based on a series of benchmark analyses, Black individuals were overrepresented in Indiana’s prisons and jails while White individuals were underrepresented in the incarceration population [59]. In 2015, 38.2% of individuals who were released in 2012 from the Indiana Department of Correction (IDOC) returned to prison. The recidivism rates for Black and White individuals were comparable, 40.2% and 38.2%, respectively [60]. Over $3,000,000 of state funds in 2015 were allocated to local prevention, treatment, and criminal justice programs in Indiana, such as indigent treatment services, problem-solving courts, jail treatment programs, and evidence-based substance use prevention programs. These monies are critical to local jurisdictions because they are often the only funds received by communities to prevent and reduce behavioral health conditions [61]. This is important in the context of the current study, as will be discussed subsequently, as Indiana stakeholders worked closely with, and supported, community-based treatment services.

In 2015, the Indiana General Assembly passed House Enrolled Act 1006, which created the Forensic Treatment Services Grant Program through the Indiana Family and Social Services Administration’s (FSSA) Division of Mental Health and Addiction (DMHA). This grant program funded the statewide Indiana Recovery Works program; a voucher-based funding program that allows criminal-legal agencies to facilitate the referral process for uninsured or underinsured Indiana adults to behavioral health service providers certified by the DMHA. Upon the start of an episode of care, providers may bill services immediately to the Recovery Works program. Eligible clients must (1) be over the age of 18, (2) be a resident of Indiana, (3) not have a taxable income that exceeds 200% of the federal income poverty level, and (4) have entered the criminal-legal system with a current felony-level charge or prior felony conviction. Both the criminal-legal agency and service provider are responsible for verifying eligibility [62].

### Data sources

We acquired secondary data from DMHA on individuals who were referred to and enrolled in the Recovery Works program between October 2015 and March 2018 (*n* = 36,718). Specifically, data were drawn from the Data Assessment Registry Mental Health and Addiction (DARMHA), which is a data system used by Recovery Works’ providers to collect client metrics for the duration of the episode of care. Through DARMHA, we procured records collected during the start of an episode of care that contained client demographics, diagnostic metrics, and baseline data from the Adult Needs and Strengths Assessment (ANSA). ANSA is a data collection tool used by clinicians upon the beginning and completion of an episode of care. The ANSA integrates data from whatever sources are available to clinicians administering the tool. Research has established evidence for the reliability of the ANSA as a communimetric^1^ measurement tool, with results indicating that the ANSA is reliable at the item level [63, 64]. All data sources included unique numeric identifiers, which facilitated the merging of these data across sources.

The sampling frame included adults with a prior or current felony-level charge who were admitted into the Recovery Works program, started an episode of care in the community, and were administered an initial behavioral health assessment. We adopted five a priori inclusion criteria to guide our approach for including clients in the current study: (1) an episode of care could be linked to a Recovery Works program enrollment date, (2) engaged in an episode of care (3) unique clients, (4) presence of a behavioral health disorder, and (5) identified race as Black or White. First, Recovery Works program enrollment was used to determine an episode of care occurred while enrolled in the program. Because many clients had recurrent episodes of care with providers prior to their initial enrollment in Recovery Works, we selected the episode of care that was no more than 31 days before a client’s enrollment. Second, all clients had to have a behavioral health assessment tied to an episode of care to indicate that they had officially started (i.e., engaged in) an episode of care. Because clients are often interviewed over multiple visits, we allowed assessments to be administered within 31 days of starting an episode of care. Third, clients could only be represented once in the sample. If a client had multiple episodes of care after enrollment between October 2015 and March 2018, we selected the first episode for inclusion in the sample. Fourth, clients needed to have been diagnosed with a substance use and/or mental health disorder. Fifth, a subsample of clients who identified as Black or White were drawn from the larger extract. Due to the nature of administrative data, missing values on covariates were present within the dataset. In total, 775 out of 16,849 records (4.6%) were incomplete. Missing cells across the entire data matrix represented less than one percent of the data, and covariates that had missingness were missing four or fewer percent of their cases.^2^ Following the application of our inclusion criteria, we used multiple imputation by chained equations. [65, 66] to handle covariates with missing data (final *N* = 16,849) and set our *m* at 5 imputations given the relatively low proportion of missing information [67]. All results are derived from pooled parameter estimates across all imputed datasets [68].

### Sample

The sample comprised 16,849 Recovery Work clients who were primarily White (*M* = 0.832, 95% confidence interval [CI] = 0.827 to 0.838) versus Black (*M* = 0.168, 95% CI = 0.162 to 0.173) and mostly male (*M* = 0.699, 95% CI = 0.692 to 0.706). Clients were an average age of 34.92 years (*SE* = 0.08) when admitted into the Recovery Works program and had an average of 1.37 (*SE* = 0.02) prior substance use episodes. A small proportion of clients had served in the military (*M* = 0.039, 95% CI = 0.036 to 0.042).

### Measures

#### Demographic characteristics

Demographic variables included *age* (continuous), *prior substance use episodes* (count), *sex* (female; male), *diagnosis* (substance use only; mental health only; co-occurring), *military service* (yes; no), and *race* (Black; White). Age, prior substance use episodes, sex, diagnosis, and military service were used as covariates in the analyses, and race was the primary variable of interest. Due to small samples sizes, clients who identified as a race other than Black or White were removed from the analysis. Clinicians used the Diagnostic and Statistical Manual of Mental Disorders 5^th^ edition (DSM-5) diagnostic criteria or the International Classification of Diseases 10^th^ revision (ICD-10) codes to classify a qualifying diagnosis. As such, clients had clinical information on their mental health or substance use disorder but could have multiple diagnoses reported in their records (i.e., co-occurring mental health and substance use disorder). Accordingly, diagnosis was recoded to account for single or co-occurring disorder.

#### Adult needs and strengths assessment

The primary outcome measure was clinician-rated *suicide risk* (evidence found; no evidence found), which was operationalized using baseline ANSA assessments [69]. The ANSA is a communimetric measurement tool that gauges progress throughout a client’s treatment on the needs and strengths of the individual across various domains. It is designed to support service/treatment planning, monitor progress, and evaluate and improve community-based services. Items are organized around six underlying community and psychiatric functioning domains to allow for ease of comprehension by clinicians during treatment referral and planning: life functioning domain (17 items), strengths domain (12 items), acculturation domain (4 items), behavioral health needs domain (10 items), risk behaviors domain (8 items), and caregiver strengths and needs domain (6 items). Additional domains can be assessed based on how clients respond to certain questions (e.g., criminogenic risk). Generally speaking, items are rated by clinicians on a 4-point scale with zero indicating no evidence of a dangerous or disabling need to three indicating a dangerous or disabling need. Items relating to strength assign values of zero (i.e., a critical strength identified) to three (i.e., no strength identified).

The risk of suicide item in the ANSA risk behavior domain was used as our dependent variable. This single item measure of clinician-rated suicide risk indicated the presence of one’s overt or covert thoughts and behaviors at attempting to die by suicide. Clinicians assigned ratings on a four-point scale (0 = *no evidence of suicide risk*; 1 = *history of suicide risk but no recent ideation or gesture during the past 30 days*; 2 = *recent suicidal ideation or gesture but not in the past 24 h*; 3 = *current ideation and intent or command hallucinations that involve self-harm*). Ratings were dichotomized to create a measure of evidence of suicide risk, indicated by a rating of one or above.

*Self-harm* (evidence found; no evidence found) was used as a covariate, which was a single item measure of self-injurious behaviors in the ANSA risk behavior domain that indicated repetitive physically harmful behavior that generally served a self-soothing functioning with the client. Clinicians assigned ratings on a four-point scale (0 = *no evidence of any forms of intentional self-injury*; 1 = *history of intentional self-injury but none evident in the past 30 days*; 2 = *engaged in self-injury that does not require medical attention*; 3 = *engaged in intentional self-injury that requires medical attention*). Ratings were dichotomized to create a measure of evidence of self-harm, indicated by a rating of one or above.

Additional covariates related to community and psychiatric functioning at Recovery Works entry were operationalized using the *life functioning* (continuous), *strengths* (continuous), *acculturation* (continuous), *behavioral health needs* (continuous), and *criminogenic risk* (continuous) domains of the ANSA. The life functioning domain is a 17-item scale (α = 0.80), measuring daily activities and skills found in the lives of individuals and their families (e.g., employment functioning, social functioning, residential stability). Comprised of 12 items, the strengths domain (α = 0.83) measures the strengths of clients’ social capital and community connectedness, such as social connectedness, education, and family strengths. The acculturation domain (α = 0.65) uses four items to capture linguistic or cultural barriers experienced in the community for which service providers need to make accommodations (e.g., language barriers, cultural stress, cultural identity). The behavioral health needs domain (α = 0.77) is a 10-item scale, covering a variety of mental health conditions and antisocial behaviors (e.g., depression, interpersonal problems, substance use). To measure criminogenic risk (α = 0.80), clinicians rated clients on 8 items that measure static and dynamic risk factors (e.g., seriousness of criminal behavior, number of prior arrests, criminal social networks). The items within each domain were combined into prorated scale scores (Range: 0–3).^3^ Higher scores on the life functioning, acculturation, behavioral health needs, and criminogenic risk scales represent more dangerous or disabling needs. Higher scores on the strengths scale signify less accessible or useful strengths found in the lives of clients.

### Analytic strategy

We conducted all analyses in Stata 15. First, descriptive statistics were conducted on all study variables to assess response distributions overall and by race categories. Measures of age, prior substance use episode, acculturation, and behavioral health needs then underwent natural log or inverse hyperbolic sine transformations to normalize skewed univariate distributions [70, 71]. Second, we conducted bivariable statistics between Black and White clients. We report the associated effect size estimates in text (i.e., Cramer’s *V*, Cohen’s *d*). Cramer’s V estimates of 0.10, 0.30, and 0.50 represent small, medium, and large effect sizes, respectively [72]. In terms of *d,* Cohen suggested corresponding estimates of 0.20, 0.50, and 0.80 indicate small, medium, and large effect sizes, respectively [72]. Third, we conducted multivariable logistic regression models separately for each racial group to model suicide risk. Fourth, we conducted hierarchical logistic regression analysis to test for evidence of between-group effects on correlates of suicide risk. Specifically, we employed nested logistic regression models to test for evidence of differences in covariates across Black and White clients. In a main-effects only model (Block 1), we included race and the remaining covariates as independent predictors of suicide risk. Block 2 included these main effects as well as added interaction terms (i.e., race X covariate). Interaction terms that were constructed with continuous and count variables were centered at the mean [73]. The F-test compared the two nested blocks where the null hypothesis is that the coefficients on the interaction terms in Block 2 are all equal to zero.

## Results

### Descriptives

Descriptive statistics for the sample overall and by race are presented in Table 1. Roughly one in every five clients presented evidence of suicide risk (*M* = 0.193, 95% CI = 0.187 to 0.199)*.* A majority of clients had a primary substance use diagnosis (*M* = 0.529, 95% CI = 0.521 to 0.536), followed by a co-occurring diagnosis (*M* = 0.417, 95% CI = 0.410 to 0.425) and a primary mental health diagnosis (*M* = 0.054, 95% CI = 0.050 to 0.057). A small proportion of clients showed evidence of self-harm behaviors (*M* = 0.106, 95% CI = 0.101 to 0.110).

**Table 1:** Descriptive statistics overall and by race

Variable	Overall *N* = 16,849	Race		Comparison Test		
		Black *n* = 2,827	White *n* = 14,022			
	*M (SE)*	*M (SE)*	*M (SE)*	Test statistic	*df*	*p*
Age	34.918 (0.08)	36.366 (0.21)	34.626 (0.08)	-6.41^a^	3,782.55	< .001
Prior substance use episode	1.369 (0.02)	1.233 (0.05)	1.396 (0.02)	11.29^c^	—	< .001
Life functioning	0.902 (< 0.01)	0.762 (0.01)	0.931 (< 0.01)	18.43^b^	16,847.00	< .001
Strengths	1.616 (< 0.01)	1.588 (0.01)	1.621 (0.01)	2.55^a^	3,913.05	.011
Acculturation	0.015 (< 0.01)	0.028 (< 0.01)	0.013 (< 0.01)	-9.86^c^	—	< .001
Behavioral health needs	0.801 (< 0.01)	0.681 (0.01)	0.826 (< 0.01)	16.34^a^	3,909.27	< .001
Criminogenic risk	1.094 (< 0.01)	1.115 (0.01)	1.090 (< 0.01)	-2.31^a^	4,161.62	.021
Race						
Black	0.168 (< 0.01)	—	—			
White	0.832 (< 0.01)	—	—			
Diagnosis				47.57^d^	2.00	< .001
Co-occurring	0.417 (< 0.01)	0.370 (0.01)	0.427 (< 0.01)			
Mental health only	0.054 (< 0.01)	0.073 (< 0.01)	0.050 (< 0.01)			
Substance use only	0.529 (< 0.01)	0.556 (0.01)	0.523 (< 0.01)			
Sex				344.33^d^	1.00	< .001
Female	0.301 (< 0.01)	0.155 (0.01)	0.330 (< 0.01)			
Male	0.699 (< 0.01)	0.845 (0.01)	0.670 (< 0.01)			
Military service				0.74^d^	1.00	0.39
Yes	0.039 (< 0.01)	0.042 (< 0.01)	0.039 (< 0.01)			
No	0.961 (< 0.01)	0.958 (< 0.01)	0.961 (< 0.01)			
Self-harm				107.42^d^	1.00	< .001
Evidence found	0.106 (< 0.01)	0.051 (< 0.01)	0.117 (< 0.01)			
No evidence found	0.894 (< 0.01)	0.949 (< 0.01)	0.883 (< 0.01)			
Suicide Risk				117.66^d^	1.00	< .001
Evidence found	0.193 (< 0.01)	0.120 (0.01)	0.208 (< 0.01)			
No evidence found	0.807 (< 0.01)	0.880 (0.01)	0.792 (< 0.01)			

^a^ unequal variance *t*-test. ^b^ equal variance *t*-test. ^c^ Wilcoxon rank-sum test. ^d^ Chi-Square test. Proportions may not sum to 1.0 due to rounding

### Bivariable comparisons

We examined bivariable comparisons between race, covariates, and suicide risk. The bivariable comparisons indicated that Black and White clients diverged significantly from one another on most variables. Black clients were found to possess significantly lower proportions of suicide risk relative to White clients, *X*^2^(1) = 117.66, *p* < 0.001, Cramer’s *V* = -0.08. Compared to White clients, Black clients were significantly older (*t*[3782.55] = -6.41, *p* < 0.001, Cohen’s *d* = -0.14) and had higher scores in both the criminogenic risk domain (*t*[4161.62] = -2.31, *p* = 0.021, Cohen’s *d* = -0.05) and the acculturation domain (*z* = -9.86, *p* < 0.001, Cohen’s *d* = -0.16). Larger proportions of Black clients had a substance use or mental health diagnosis compared to White clients. Alternatively, more White clients had a co-occurring diagnosis compared to Black clients, *X*^2^(2) = 47.57, *p* < 0.001, Cramer’s *V* = 0.05. A greater proportion of White clients were female relative to Black clients, *X*^2^(1) = 344.33, *p* < 0.001, Cramer’s *V* = -0.14. White clients had a larger ratio of presenting evidence of self-harm than Black clients, *X*^2^(1) = 107.42, *p* < 0.001, Cramer’s *V* = -0.08. In relation to Black clients, White clients had more prior substance use episodes (*z* = 11.29, *p* < 0.001, Cohen’s *d* = 0.20) and had higher scores on the life functioning domain (*t*[16847] = 18.43, *p* < 0.001, Cohen’s *d* = 0.38), strengths domain (*t*[3913.05] = 2.55, *p* = 0.011, Cohen’s *d* = 0.05), and behavioral health needs domain (*t*[3909.27] = 16.34, *p* < 0.001, Cohen’s *d* = 0.35). There were no statistically significant differences between Black and White clients in prior military service.

### Multivariable models

Table 2 presents results of multivariable logistic regression models of suicide risk separately for each racial group. While a co-occurring diagnosis relative to a substance use diagnosis (OR = 1.91, 95% CI = 1.35 to 2.70, *p* < 0.001) was an important predictor of suicide risk in Model 1, the behavioral health needs domain (OR = 9.84, 95% CI = 5.43 to 17.83), self-harm (OR = 8.00, 95% CI = 5.25 to 12.18), the life functioning domain (OR = 2.08, 95% CI = 1.38 to 3.16), and a mental health diagnosis relative to a substance use diagnosis (OR = 1.95, 95% CI = 1.19 to 3.20) were the strongest unique predictors of suicide risk for Black clients, *p*s ≤ 0.008. Age, military service, prior substance use episodes, the strengths domain, and the acculturation domain were not significantly associated with suicide risk in Black clients, *p*s ≥ 0.057. In Model 2, the behavioral health needs domain (OR = 8.68, 95% CI = 7.05 to 10.70), self-harm (OR = 5.44, 95% CI = 4.81 to 6.16), and a mental health diagnosis (OR = 1.94, 95% CI = 1.56 to 2.42) or a co-occurring diagnosis (OR = 1.75, 95% CI = 1.57 to 1.94) relative to a substance use diagnosis were the strongest unique predictors of suicide risk in White clients, *p*s < 0.001, followed by the life functioning domain (OR = 1.45, 95% CI = 1.25 to 1.70, *p* < 0.001). Age, the strengths domain, and the acculturation domain were not significantly associated with suicide risk in White clients, *p*s ≥ 0.131. Seven correlates uniquely contributed to the prediction of suicide risk in both models: mental health diagnosis (OR range: 1.94–1.95), co-occurring diagnosis (OR range: 1.75–1.91), self-harm (OR range: 5.44–8.00), female (OR range: 1.16–1.51), life functioning (OR range: 1.45–2.08), behavioral health needs (OR range: 8.68–9.84), and criminogenic risk (OR range: 0.61–0.69), *p*s ≤ 0.013.

**Table 2:** Logistic regression models predicting suicide risk, by race

Predictor	Suicide Risk									
	**Black** Model 1 *n* = 2,827					**White** Model 2 *n* = 14,022				
	Estimate	*SE*	t	OR	95% CI	Estimate	*SE*	t	OR	95% CI
Diagnosis ^(Substance use only)^										
Mental health only	0.67	0.25	2.67**	1.95	[1.19, 3.20]	0.66	0.11	5.90***	1.94	[1.56, 2.42]
Co-occurring	0.65	0.18	3.65***	1.91	[1.35, 2.70]	0.56	0.05	10.22***	1.75	[1.57, 1.94]
Self-harm ^(No evidence found)^	2.08	0.21	9.69***	8.00	[5.25, 12.18]	1.69	0.06	26.82***	5.44	[4.81, 6.16]
Age	0.06	0.23	0.25	1.06	[0.67, 1.67]	0.14	0.09	1.51	1.15	[0.96, 1.37]
Female ^(Male)^	0.41	0.17	2.49*	1.51	[1.09, 2.09]	0.15	0.05	2.89**	1.16	[1.05, 1.28]
Military service ^(No)^	0.58	0.31	1.91	1.79	[0.98 3.27]	0.26	0.12	2.18*	1.30	[1.03, 1.64]
Prior substance use episodes	-0.01	0.09	-0.08	0.99	[0.84, 1.18]	0.09	0.03	3.02**	1.10	[1.03, 1.16]
Life functioning	0.73	0.21	3.46**	2.08	[1.38, 3.16]	0.37	0.08	4.73***	1.45	[1.25, 1.70]
Strengths	0.19	0.13	1.39	1.21	[0.93, 1.57]	0.05	0.05	1.06	1.05	[0.96, 1.16]
Acculturation	-0.64	0.49	-1.31	0.53	[0.20, 1.37]	-0.07	0.25	-0.30	0.93	[0.57, 1.52]
Behavioral health needs	2.29	0.30	7.54***	9.84	[5.43, 17.83]	2.16	0.11	20.29***	8.68	[7.05, 10.70]
Criminogenic risk	-0.50	0.14	-3.44**	0.61	[0.46, 0.81]	-0.37	0.05	-7.45***	0.69	[0.63, 0.76]

For categorical variables, reference group indicated in parentheses. *CI:* confidence interval for odds ratio

^*^p < .05. **p < .01. ***p < .001. (two-tailed)

Table 3 presents results of multivariable logistic regression models of suicide risk for the sample overall, adding race as a covariate. Black clients were significantly less likely (OR = 0.75, 95% CI = 0.65 to 0.86, *p* < 0.001) than White clients to be at risk of suicide. In Block 2, we examined whether race moderated the effect of covariates on suicide risk. Together, the addition of the interaction terms did not contribute to a significant improvement in model fit over Block 1, *p* = 0.107. We observed no evidence of between-group effects on correlates of suicide risk.

**Table 3:** Logistic regression models predicting suicide risk

Predictor	Suicide Risk				
	Estimate	*SE*	t	OR	95% CI
**Block 1**					
Black ^(White)^	-0.29	0.07	-4.14***	0.75	[0.65, 0.86]
Diagnosis ^(Substance use only)^					
Mental health only	0.67	0.10	6.48***	1.96	[1.60, 2.41]
Co-occurring	0.57	0.05	10.69***	1.76	[1.59, 1.96]
Self-harm ^(No evidence found)^	1.72	0.06	28.45***	5.61	[4.98, 6.32]
Age	0.14	0.08	1.62	1.15	[0.97, 1.35]
Female ^(Male)^	0.17	0.05	3.45**	1.18	[1.07, 1.30]
Military service ^(No)^	0.30	0.11	2.70**	1.35	[1.09, 1.68]
Prior substance use episodes	0.08	0.03	2.83**	1.08	[1.03, 1.15]
Life functioning	0.42	0.07	5.65***	1.52	[1.31, 1.76]
Strengths	0.06	0.05	1.39	1.07	[0.97, 1.17]
Acculturation	-0.18	0.22	-0.81	0.84	[0.54, 1.29]
Behavioral health needs	2.19	0.10	21.88***	8.97	[7.37, 10.92]
Criminogenic risk	-0.38	0.05	-8.30***	0.68	[0.62, 0.75]
**Block 2**					
Black					
X Diagnosis – Mental health only	0.01	0.27	0.02	1.01	[0.59, 1.70]
X Diagnosis – Co-occurring	0.09	0.18	0.48	1.09	[0.76, 1.56]
X Self-harm	0.39	0.22	1.72	1.47	[0.95, 2.28]
X Age	-0.08	0.25	-0.32	0.92	[0.57, 1.51]
X Female	0.27	0.17	1.55	1.31	[0.93, 1.84]
X Military service	0.32	0.33	0.99	1.38	[0.73, 2.63]
X Prior substance use episodes	-0.10	0.09	-1.05	0.91	[0.76, 1.09]
X Life functioning	0.36	0.23	1.59	1.43	[0.92, 2.23]
X Strengths	0.14	0.14	0.95	1.14	[0.86, 1.52]
X Acculturation	-0.57	0.55	-1.04	0.57	[0.19, 1.66]
X Behavioral health needs	0.13	0.32	0.39	1.13	[0.60, 2.13]
X Criminogenic risk	-0.12	0.15	-0.87	0.88	[0.65, 1.18]
	*F* (12, 177,605.6^a^) = 1.53, *p* = .107				

*N* = 16,849. For categorical variables, reference group indicated in parentheses. All model terms from Block 1 were included in Block 2; however, only unique terms are shown. *CI:* confidence interval for odds ratio. ^a^ Residual degrees of freedom

^**^p < .01. ***p < .001. (two-tailed)

## Discussion

Against a backdrop of rising suicide rates within criminal-legal populations, risk factors for suicide can be amplified by barriers to successful community reentry. Our study extends the current literature by examining baseline evaluations of clinician-rated suicide among adults recently involved in criminal-legal systems but additionally have a diagnosed behavioral health disorder, which also places them at an elevated risk of suicide relative to the general population [74]. Among this population, we examined co-occurring mental health and substance use disorder and found significantly higher suicide risk relative to those with substance use alone. This is consistent with prior research and is particularly important to consider in treatment settings [22, 74, 75]. Our study also identified a number of additional factors that were associated with suicide risk, which were generally consistent with other findings [6, 9, 76, 77]. One notable exception was the negative association between criminogenic risk and suicide [15, 18]. Given the criminogenic risk domain was rated by clinicians on 8 items that measure static and dynamic risk factors, our indicator may have captured a different construct than used in prior studies and warrants further investigation.

Our findings showed that Black clients had a lower likelihood of suicide risk in relation to White clients, which is consistent with prior findings [1, 3]. Similar to biases found within clinical prediction models of suicide [38], biases on the part of the clinician (e.g., assuming Black clients have a lower suicide risk based on historical trends) may be contributing to the lower likelihood of clinician-rated suicide risk among Black clients. Suicide misclassification is more likely to occur with Black individuals relative to White individuals [78], which may result from insufficient documentation of behavioral health history [79]. Research shows that perceived discrimination in behavioral health treatment contributes to fragmented episodes of care for Black individuals [80]. Recovery Works’ clinicians score the ANSA based on whatever sources are available to them, but discrepant behavioral health documentation due to known barriers to treatment among Black individuals involved in criminal-legal systems may limit a clinician’s ability to accurately assess suicide risk. This speculation warrants future research.

Our findings also suggest some of the unique markers of suicide risk in adults with criminal-legal contact may actually represent more general risk factors for this population given they operate similarly across both groups. For example, we identified seven consistent correlates of suicide risk for both Black and White clients in the race-specific analyses that included mental health diagnosis, co-occurring diagnosis, self-harm, female, life functioning, behavioral health needs, and criminogenic risk. However, as in other studies [81, 82], we found that a greater number of prior substance abuse episodes and military service were uniquely associated with a higher likelihood of suicide risk for White and not Black clients. While bivariable comparisons and race-specific analyses suggested potential differences by race in some of the correlates of suicide risk, we found no evidence of statistically significant between-group effects in our multivariable model with added interactions terms. These non-significant interactions between race and risk factors for suicide may be the result of the clinician-rated measures we used or driven by our use of a treatment-engaged sample. Risk factors in the absence of treatment could present differently, particularly given disparities in behavioral health treatment utilization between Black and White populations [35–37].

Even in the presence of shared risk factors, prior research on culture and suicide reinforce the reality that suicide risk involves a complex interaction between cultural forces, informal social networks, personal situations, and predispositions [83–86]. As such, treatment services or interventions should not exist in a vacuum. Our findings do not negate the importance of providers and criminal-legal personnel incorporating knowledge on group-specific dimensions for suicide risk into programming for adults with criminal-legal involvement to yield more inclusive approaches to treatment services and interventions [87–89]. Understanding both cultural variations and shared factors in risk management of suicide may enhance therapeutic relationships and inform risk evaluations for high-needs populations [90, 91]. Adults with behavioral health conditions in criminal-legal systems have multifaceted needs that require more comprehensive services and individualized responses to each client’s circumstance [92]. Positive psychiatric and community functioning outcomes resulting from diverting individuals with behavioral health disorders away from criminal-legal systems are relatively well-documented [93–95]. In the absence of diversion, providing timely linkages upon release from incarceration to affordable behavioral health treatments are essential to psychiatric rehabilitation and community reintegration [96, 97]. Yet, criminal-legal contacts that are influenced by bias, racism, and discrimination contribute to diminished utilization of services and fragmented episodes of care for Black persons in criminal-legal populations.

It is also crucial to remember that the sample of people with behavioral health disorders examined in this study were referred by someone in the criminal-legal system where research nationally shows Black people experiencing harsher punishments across nearly every encounter [98]. Recovery Works is one of the first statewide, client centered recovery models that offers access to a mix of clinical and wraparound support services through referrals and an integrated system of care for felony-level adults. Investigations into how clients are referred and connected to needed care and where racial disparities might exist in referrals are critically important and are being examined through a separate scope of work. However, the administrative data from this novel program have allowed us to advance the literature by examining the race-specific correlates of suicide risk for adults with criminal-legal involvement seeking behavioral health treatment in the community.

As noted above, the Recovery Works program represents a recovery-oriented system of care, which is based on the Substance Abuse Mental Health Services Administration’s (SAMHSA) conceptualization of recovery. Designated providers employ client-centered techniques (e.g., motivational interviewing, trauma-informed care, harm reduction strategies) in developing and assessing a client’s recovery plan. Research on the effects of these approaches suggest improvements in treatment outcomes across diverse clinical settings [99–101], including suicide risk [102, 103]. Suicidality and traumatic stress co-occur in criminal-legal populations [104, 105], which underscores the need for incorporating client-centered principles—such as trauma-informed care—into the evaluation and management of suicide risk. Yet, research on the effect of Recovery Works’ components and content of services on criminal-legal populations who are at risk for suicide—with attention to cultural variations in suicide risk—is needed. This might include assessing the trainings and procedures that prospective agencies undergo to become designated treatment providers or obtaining data on the attributes of providers (e.g., specific provider qualifications, program standards, target population, provided services). Relatedly, behavioral health assessments at the start of an episode of care influence service provision. The accuracy of assessments by clinicians represents an important research avenue to address disparities in need and access.

## Limitations

The results should be interpreted with limitations in mind. First, the cross-sectional design of this study limited our ability to establish temporal order or infer causal relationships. Second, the nature of the study limited us to utilizing administrative data collected by DMHA-certified providers, which prevented us from collecting data on referral sources, provider characteristics, and individual-and structural-level risk and protective factors that may have directly or indirectly predicted suicide risk (e.g., personality traits, disabilities, experiences related to victimization and trauma, access to firearms, community disadvantage, quality of service delivery). Relatedly, a single item of clinician-rated suicide risk may not have adequately measured the construct compared to a self-report, multi-item scale. Studies using similar, single item measures of suicide risk from larger inventories, however, were found to be valid [77, 106, 107]. Third, we obtained data representing a large, community-based sample of adults with criminal-legal contact who engaged in behavioral health treatment through a single statewide funding program. Future research should examine similar research questions in other jurisdictions to determine the generalizability of the findings.

Limitations notwithstanding, our study improves on prior research in several respects. We implemented race-specific analyses to explicitly examine potential differences in suicide risk across White and Black adults with criminal-legal involvement, which is a critical step to identifying and targeting interventions toward at-risk individuals who are involved in the criminal-legal system and safely managing those at greatest risk for suicide. We also modeled and adjusted for co-occurring substance use and mental health disorders, which has been understudied in the field. Given the prevalence of comorbidities in the criminal-legal system, models that account for this risk factor may achieve more accurate results. Finally, this study involved a unique sample: a large, community-based sample of adults with criminal-legal contact who engaged in behavioral health treatment (*N* = 16,849), which is considerably larger and more representative than previous studies in this population [22, 42].

Our results provide several directions for future research. This investigation to our knowledge contributes to a limited body of knowledge that examines the racial differences in suicide risk among a community-based sample of felony-level adults with behavioral health disorders in the criminal-legal system; replication and further research is needed. Our study suggested factors precipitating clinician-rated suicide risk did not vary across race. The role of risk and protective factors on suicide risk may differ as a function of the operationalization of measures, the sample, and the criminal-legal setting. The evolving sociopolitical climate around race and the criminal-legal system also underscores the need for continued dialogue and research on how justice involvement might amplify suicide risk. Lastly, we note the important overlaps between criminal-legal involvement, suicide risk, and the current opioid epidemic in the United States. There have been a number of changes in how local law enforcement agencies have addressed substance use [108], and there have been increases in opioid-related overdose deaths [109] and impairments with community functioning [110] among Black individuals, in particular. Unfortunately, this cross-sectional baseline study only assessed crude measures of substance use and could not examine comprehensive treatment-or recovery-oriented measures. Future research should integrate robust measures of self-reported primary drug use or objective drug test results which would provide a deeper understanding of how drug use influences suicide risk in criminal-legal populations.

## Conclusion

Risk and protective factors for suicide in the general population are well-documented, but there is limited evidence on how important these factors are within criminal-legal populations in general and behavioral health populations in a criminal-legal system context in particular. The current findings suggest that adults with behavioral health disorders and recent criminal-legal system contact experience similar risk factors for suicide as the general population. Black individuals, in particular, are at lower risk for suicide overall. Contrary to prior research demonstrating racial differences in correlates of suicide risk, the current study revealed similarities in correlates of suicide risk across Black and White adults in criminal-legal systems. However, it remains to be seen whether these findings generalize to other jurisdictions and other behavioral health populations in criminal-legal systems. Future research and replications should address the limitations noted in this study. Given documented racial disparities in behavioral health services and criminal-legal systems, research that integrates shared risk factors and known culture-specific influences on suicide risk may advance suicide prevention efforts.

## Data Availability

The measures section provides references to where all materials used in this article can be obtained from. The analysis script for the present research can be obtained from the authors upon request.

## Abbreviations

DSM-IV: The fourth edition of the Diagnostic and Statistical Manual of Mental Disorders
IDOC: Indiana Department of Correction
FSSA: Family and Social Services Administration
DMHA: Division of Mental Health and Addiction
DARMHA: Data Assessment Registry Mental Health and Addiction
ANSA: Adult Needs and Strengths Assessment
CI: Confidence interval
DSM-5: The fifth edition of the Diagnostic and Statistical Manual of Mental Disorders
ICD-10: The tenth revision of the International Classification of Diseases
SAMHSA: Substance Abuse Mental Health Services Administration
OR: Odds ratio
*SE*: Standard error
*M*: Mean

## Footnotes

1 Communimetrics represents a communication theory of measurement in human service settings. Ease of interpretation and utility underscore design and selection of communimetric measures, without compromising replicability and accuracy. The primary reason to measure within a communimetrics approach is to better assist providers in the translation of assessment information into practical service planning for their clients [111].

2 Percent missing of each covariate: 4.02% for diagnosis, 0.59% for criminogenic risk, and 0.04% for age. All other covariates had no missing data.

3 Scale scores were prorated by averaging the nonmissing items for cases that had no more than 20% of the item responses missing within each ANSA domain [112]. The life functioning domain had 96.4% of complete data, and 100% of cases were scoreable. The strengths domain had 95.8% of complete data, and 100% of cases were scoreable. The criminogenic risk domain had 99.4% of complete data, and 99.4% of cases were scoreable. The remaining domains had 100% of complete data, thus, 100% of cases were scoreable.

## References

[1] Kochanek KD, Murphy SL, Xu J, Arias E. Deaths: Final Data for 2017 [Internet]. Hyattsville, MD: National Center for Health Statistics, Centers for Disease Control and Prevention; 2019 [cited 2021 Jul 21]. Report No.: National Vital Statistics Reports; Vol 68 No 9. Available from: https://www.cdc.gov/nchs/data/nvsr/nvsr68/nvsr68_09-508.pdf

[2] Centers for Disease Control and Prevention. Preventing suicide [Internet]. Washington, D.C.; 2018 [cited 2021 Jul 21]. Available from: https://www.cdc.gov/violenceprevention/pdf/suicide-factsheet.pdf

[3] Stone DM, Simon TR, Fowler KA, Kegler SR, Yuan K, Holland KM (2018). Vital signs: Trends in state suicide rates — United States, 1999–2016 and circumstances contributing to suicide — 27 states, 2015. Morb Mortal Wkly Rep.

[4] Hedegaard H, Curtin SC, Warner M. Increase in suicide mortality in the United States, 1999–2018 [Internet]. Hyattsville, MD: National Center for Health Statistics, Centers for Disease Control and Prevention; 2020 [cited 2021 Jul 21]. Report No.: NCHS Data Brief, No. 362. Available from: https://www.cdc.gov/nchs/data/databriefs/db362-h.pdf

[5] Substance Abuse and Mental Health Services Administration. Key substance use and mental health indicators in the United States: Results from the 2018 National Survey on Drug Use and Health [Internet]. Rockville, MD: Center for Behavioral Health Statistics and Quality, Substance Abuse and Mental Health Services Administration; 2019 [cited 2021 Jul 21]. Report No.: (HHS Publication No. PEP19-5068, NSDUH Series H-54. Available from: https://www.samhsa.gov/data/sites/default/files/cbhsq-reports/NSDUHNationalFindingsReport2018/NSDUHNationalFindingsReport2018.pdf

[6] Franklin JC, Ribeiro JD, Fox KR, Bentley KH, Kleiman EM, Huang X (2017). Risk factors for suicidal thoughts and behaviors: A meta-analysis of 50 years of research. Psychol Bull.

[7] Yoshimasu K, Kiyohara C, Miyashita K, Stress Research Group of the Japanese Society for Hygiene (2008). Suicidal risk factors and completed suicide: Meta-analyses based on psychological autopsy studies. Environ Health Prev Med.

[8] Demir M (2018). Gender differences in suicide rates. Forensic Science & Addiction Research.

[9] Lange S, Bagge C, Probst C, Rehm J. Proportion of individuals with past-year suicidal ideation who attempted suicide over the past 10 years in the United States, and the influence of age and sex. Crisis: J Crisis Interven Suicide Prev. 2021;42(2):152–6. 10.1027/0227-5910/a000690 32431200

[10] Bryson WC, Piel J, Thielke S (2021). Associations between parole, probation, arrest, and self-reported suicide attempts. Community Ment Health J.

[11] Carson EA, Cowhig MP. Mortality in state and federal prisons, 2001–2016 - Statistical tables [Internet]. Washington, D.C.: Bureau of Justice Statistics, Office of Justice Programs, U.S. Department of Justice; 2020 [cited 2021 Jul 21]. Available from: https://www.bjs.gov/content/pub/pdf/msfp0116st.pdf

[12] DeCou CR, Lynch SM, DeHart DD, Belknap J (2016). Evaluating the association between childhood sexual abuse and attempted suicide across the lifespan: Findings from a nationwide study of women in jail. Psychol Serv.

[13] Waesche MC, Clark CB, Cropsey KL (2016). The connection between thwarted belongingness, alcohol consumption, suicidal, and homicidal ideation in a criminal justice sample. J Addict Med.

[14] You S, Swogger MT, Cerulli C, Conner KR (2011). Interpersonal violence victimization and suicidal ideation: An examination in criminal offenders. Crisis: J Crisis Interven Suicide Prev.

[15] Blaauw E, Kerkhof AJFM, Hayes LM (2005). Demographic, criminal, and psychiatric factors related to inmate suicide. Suicide Life Threat Behav.

[16] Folk JB, Loya JM, Alexoudis EA, Tangney JP, Wilson JS, Barboza SE (2018). Differences between inmates who attempt suicide and who die by suicide: Staff-identified psychological and treatment-related risk factors. Psychol Serv.

[17] Magaletta PR, Patry MW, Wheat B, Bates J (2008). Prison inmate characteristics and suicide attempt lethality: An exploratory study. Psychol Serv.

[18] Webb RT, Qin P, Stevens H, Mortensen PB, Appleby L, Shaw J (2011). National study of suicide in all people with a criminal justice history. Arch Gen Psychiatry.

[19] Bronson J, Stroop J, Zimmer S, Berzofsky M. Drug use, dependence, and abuse among state prisoners and jail inmates, 2007–2009 [Internet]. Washington, D.C.: Bureau of Justice Statistics, Office of Justice Programs, U.S. Department of Justice; 2017 [cited 2021 Jul 21]. Report No.: NCJ 250546. Available from: https://www.bjs.gov/content/pub/pdf/dudaspji0709.pdf

[20] Bronson J, Berzofsky M. Indicators of mental health problems reported by prisoners and jail inmates, 2011–12 [Internet]. Washington, D.C.: Bureau of Justice Statistics, Office of Justice Programs, U.S. Department of Justice; 2017 [cited 2021 Jul 21]. Report No.: NCJ 250612. Available from: https://www.bjs.gov/content/pub/pdf/imhprpji1112.pdf

[21] The National Center on Addiction and Substance Abuse. Behind bars II: Substance abuse and America’s prison population [Internet]. New York, NY: Columbia University; 2010. Available from: https://drugfree.org/reports/behind-bars-ii-substance-abuse-and-americas-prison-population/

[22] Cardarelli R, Balyakina E, Malone K, Fulda KG, Ellison M, Sivernell R (2015). Suicide risk and mental health co-morbidities in a probationer population. Community Ment Health J.

[23] Hakansson A, Bradvik L, Schlyter F, Berglund M. Factors associated with the history of attempted suicide: A criminal justice population examined with the Addiction Severity Index (ASI). Crisis: J Crisis Interven Suicide Prev. 2010;31(1):12–21;31(1):12–21. 10.1027/0227-5910/a000008 20197253

[24] Carson EA. Prisoners in 2018 [Internet]. Washington, D.C.: Bureau of Justice Statistics, Office of Justice Programs, U.S. Department of Justice; 2020 [cited 2021 Jul 21]. Available from: https://www.bjs.gov/content/pub/pdf/p18.pdf

[25] Cobbina JE (2019). Hands up, don’t shoot: Why the protests in Ferguson and Baltimore matter, and how they changed America.

[26] Kochel TR, Wilson DB, Mastrofski SD (2011). Effect of suspect race on officers’ arrest decisions. Criminology.

[27] Zeng Z. Jail inmates in 2018 [Internet]. Washington, DC: Bureau of Justice Statistics, Office of Justice Programs, U.S. Department of Justice; 2020 [cited 2021 Jul 21]. Report No.: NCJ 253044. Available from: https://www.bjs.gov/content/pub/pdf/ji18.pdf

[28] Manza J, Uggen C (2008). Locked out: Felon disenfranchisement and American democracy.

[29] Wang X, Mears DP, Bales WD (2010). Race-specific employment contexts and recidivism. Criminology.

[30] Williams JM, Wilson SK, Bergeson C (2019). “It’s hard out here if you’re a Black felon”: A critical examination of Black male reentry. The Prison Journal.

[31] Blankenship KM, del R Gonzalez AM, Keene DE, Groves AK, Rosenberg AP. Mass incarceration, race inequality, and health: Expanding concepts and assessing impacts on well-being. Soc Sci Med. 2018;215:45–52;215:45–52. 10.1016/j.socscimed.2018.08.042 PMC6324558 30205278

[32] Massoglia M (2008). Incarceration, health, and racial disparities in health. Law Soc Rev.

[33] Lowder EM, Rade CR, Desmarais SL (2018). Effectiveness of mental health courts in reducing recidivism: A meta-analysis. Psychiatr Serv.

[34] Garnick DW, Horgan CM, Acevedo A, Lee MT, Panas L, Ritter GA (2014). Criminal justice outcomes after engagement in outpatient substance abuse treatment. J Subst Abuse Treat.

[35] Cook BL, Trinh N-H, Li Z, Hou SS-Y, Progovac AM (2016). Trends in racial-ethnic disparities in access to mental health care, 2004–2012. Psychiatr Serv.

[36] Cook BL, Hou SS-Y, Lee-Tauler SY, Progovac AM, Samson F, Sanchez MJ (2019). A review of mental health and mental health care disparities research: 2011–2014. Med Care Res Rev.

[37] Koizumi N, Rothbard AB, Kuno E. Distance matters in choice of mental health program: Policy implications for reducing racial disparities in public mental health care. Adm Policy Ment Health. 2009;36(6):424–31. 10.1007/s10488-009-0233-z 19653093

[38] Coley RY, Johnson E, Simon GE, Cruz M, Shortreed SM (2021). Racial/ethnic disparities in the performance of prediction models for death by suicide after mental health visits. JAMA Psychiat.

[39] Alim TN, Graves E, Mellman TA, Aigbogun N, Gray E, Lawson W (2006). Trauma exposure, posttraumatic stress disorder and depression in an African-American primary care population. J Natl Med Assoc.

[40] Gara MA, Vega WA, Arndt S, Escamilla M, Fleck DE, Lawson WB (2012). Influence of patient race and ethnicity on clinical assessment in patients with affective disorders. Arch Gen Psychiatry.

[41] Williams DR, González HM, Neighbors H, Nesse R, Abelson JM, Sweetman J (2007). Prevalence and distribution of major depressive disorder in African Americans, Caribbean Blacks, and Non-Hispanic Whites: Results from the National Survey of American Life. Arch Gen Psychiatry.

[42] Clark CB, Li Y, Cropsey KL (2016). Family dysfunction and suicide risk in a community corrections sample. J Crisis Interven Suicide Prevent.

[43] Sirdifield C, Brooker C, Marples R (2020). Suicide and probation: A systematic review of the literature. Forensic Science International: Mind and Law.

[44] Ahuja M, Records K, Haeny AM, Gavares EM, Mamudu HM (2021). The association between experiencing police arrest and suicide ideation among emerging young adults: Does race matter?. Health Psychology Open.

[45] McCullumsmith CB, Clark CB, Perkins A, Fife J, Cropsey KL (2013). Gender and racial differences for suicide attempters and ideators in a high-risk community corrections population. Crisis: J Crisis Interven Suicide Prev.

[46] Curtin SC, Hedegaard H. Suicide rates for females and males by race and ethnicity: United States, 1999 and 2017 [Internet]. Hyattsville, MD: National Center for Health Statistics, Centers for Disease Control and Prevention; 2019 [cited 2021 Jul 21]. Available from: https://www.cdc.gov/nchs/data/hestat/suicide/rates_1999_2017.pdf

[47] Czeisler MÉ, Lane RI, Petrosky E, Wiley JF, Christensen A, Njai R (2020). Mental health, substance use, and suicidal ideation during the COVID-19 pandemic — United States, June 24–30, 2020. Morb Mortal Wkly Rep.

[48] Gomez J, Miranda R, Polanco L (2011). Acculturative stress, perceived discrimination, and vulnerability to suicide attempts among emerging adults. J Youth Adolesc.

[49] Walker RL, Wingate LR, Obasi EM, Joiner TE (2008). An empirical investigation of acculturative stress and ethnic identity as moderators for depression and suicidal ideation in college students. Cultur Divers Ethnic Minor Psychol.

[50] Compton MT, Thompson NJ, Kaslow NJ (2005). Social environment factors associated with suicide attempt among low-income African Americans: The protective role of family relationships and social support. Soc Psychiatry Psychiatr Epidemiol.

[51] Nguyen AW, Taylor RJ, Chatters LM, Taylor HO, Lincoln KD, Mitchell UA (2017). Extended family and friendship support and suicidality among African Americans. Soc Psychiatry Psychiatr Epidemiol.

[52] Hooper LM, Tomek S, Jaggers J, Idigo C, Church WT, Williams J (2017). Changes in perceived levels of environmental stress before and after a suicide attempt in Black American adolescents: A 14-year longitudinal study. J Ment Health Couns.

[53] Kaslow NJ, Sherry A, Bethea K, Wyckoff S, Compton MT, Bender Grall M (2005). Social risk and protective factors for suicide attempts in low income African American men and women. Suicide Life Threat Behav.

[54] Anglin DM, Gabriel KOS, Kaslow NJ (2005). Suicide acceptability and religious well-being: A comparative analysis in African American suicide attempters and non-attempters. J Psychol Theol.

[55] Richardson-Vejlgaard R, Sher L, Oquendo MA, Lizardi D, Stanley B (2009). Moral objections to suicide and suicidal ideation among mood disordered Whites, Blacks, and Hispanics. J Psychiatr Res.

[56] Reed DD, Stoeffler SW, Joseph R. Suicide, race, and social work: A systematic review of protective factors among African Americans. J Evid Based Soc Work. 2021;18(4):379–93. 10.1080/26408066.2020.1857317 33622190

[57] American Community Survey. ASC 5-year estimates. Table DP05 [Internet]. Washington, D.C.: U.S. Census Bureau; 2015. Available from: https://data.census.gov/cedsci/table?tid=ACSDP5Y2015.DP05&g = 0400000US18

[58] Carson EA, Anderson E. Prisoners in 2015 [Internet]. Washington, D.C.: Bureau of Justice Statistics, Office of Justice Programs, U.S. Department of Justice; 2016 [cited 2022 Jan 18]. Available from: https://bjs.ojp.gov/content/pub/pdf/p15.pdf

[59] Sakala L. Breaking down mass incarceration in the 2010 Census [Internet]. Washington, D.C.: Prison Policy Initiative; 2014 [cited 2022 Jan 25]. Available from: https://www.prisonpolicy.org/reports/rates.html

[60] Indiana Department of Correction. 2015 adult recidivism rates [Internet]. Indianapolis, IN; 2015. Available from: https://www.in.gov/idoc/data-and-statistics/statistical-data/recidivism-reports/

[61] Indiana Criminal Justice Institute. Indiana Criminal Justice Institute: 2015 annual report [Internet]. Indianapolis, IN; 2015 [cited 2022 Jan 18]. Available from: https://www.in.gov/cji/grant-opportunities/files/2015-ICJI-Annual-Report-FINAL.pdf

[62] Recovery Works. Recovery Works Policies and Procedures Manual [Internet]. Indianapolis, IN: Division of Mental Health and Addiction, Indiana Family and Social Services Administration; 2019 [cited 2021 Jul 21]. Available from: https://www.in.gov/fssa/dmha/files/RW-PPM.pdf

[63] Nelson C, Johnston M (2008). Adult Needs and Strengths Assessment-Abbreviated Referral version to specify psychiatric care needed for incoming patients: Exploratory analysis. Psychol Rep.

[64] Praed Foundation. Adult Needs and Strengths Assessment (ANSA) [Internet]. Chicago, IL; 2021 [cited 2021 Jul 21]. Available from: https://praedfoundation.org/tools/the-adult-needs-and-strengths-assessment-ansa/

[65] Royston P (2004). Multiple Imputation of missing values. Stand Genomic Sci.

[66] White IR, Royston P, Wood AM (2011). Multiple imputation using chained equations: Issues and guidance for practice. Stat Med.

[67] Graham JW, Olchowski AE, Gilreath TD (2007). How many imputations are really needed? Some practical clarifications of multiple imputation theory. Prev Sci.

[68] Rubin DB (2004). Multiple imputation for nonresponse in surveys.

[69] Praed Foundation. Adult Needs and Strengths Assessment (ANSA): Indiana ANSA manual - An information integration tool for adults with behavioral health challenges [Internet]. 2015 [cited 2021 Jul 21]. Report No.: Version 2.3. Available from: https://dmha.fssa.in.gov/darmha/Documents/ANSA_Manual_SFY2017.pdf

[70] Burbidge JB, Magee L, Robb AL (1988). Alternative transformations to handle extreme values of the dependent variable. J Am Stat Assoc.

[71] Osborne J (2010). Improving your data transformations: Applying the Box-Cox transformation. Pract Assess Res Eval.

[72] Cohen J (1988). Statistical power analysis for the behavioral sciences.

[73] Iacobucci D, Schneider MJ, Popovich DL, Bakamitsos GA (2016). Mean centering helps alleviate “micro” but not “macro” multicollinearity. Behav Res Methods.

[74] Restrepo D, Gutierrez-Ochoa N, Rodriguez-Echeverri C, Sierra-Hincapie G (2019). Suicide risk associated with dual diagnosis in general population. Addict Disord Treat.

[75] Marzano L, Hawton K, Rivlin A, Smith EN, Piper M, Fazel S. Prevention of suicidal behavior in prisons: An overview of initiatives based on a systematic review of research on near-lethal suicide attempts. Crisis: J Crisis Interven Suicide Prev. 2016;37(5):323–34. 10.1027/0227-5910/a000394 PMC5120691 27278569

[76] Dennis BB, Roshanov PS, Bawor M, ElSheikh W, Garton S, DeJesus J (2015). Re-examination of classic risk factors for suicidal behavior in the psychiatric population. J Crisis Interven Suicide Prev..

[77] Jahn DR, Muralidharan A, Drapalski AL, Brown CH, Fang LJ, Lucksted A (2018). Differences in suicide and death ideation among veterans and nonveterans with serious mental illness. Psychol Serv.

[78] Ali B, Rockett IR, Miller TR, Leonardo JB. Racial/ethnic differences in preceding circumstances of suicide and potential suicide misclassification among US adolescents. J Racial Ethn Health Disparities. 2022;9(1):296–304. 10.1007/s40615-020-00957-7 33415703

[79] Rockett IR, Wang S, Stack S, De Leo D, Frost JL, Ducatman AM (2010). Race/ethnicity and potential suicide misclassification: Window on a minority suicide paradox?. BMC Psychiatry.

[80] Mays VM, Jones A, Delany-Brumsey A, Coles C, Cochran SD (2017). Perceived discrimination in healthcare and mental health/substance abuse treatment among Blacks, Latinos, and Whites. Med Care.

[81] Britton PC, Conner KR. Suicide attempts within 12 months of treatment for substance use disorders. Suicide and Life-Threatening Behavior. 2010 Feb;40(1):14–21. 10.1521/suli.2010.40.1.14 PMC5064437 20170258

[82] Griffith J (2012). Suicide in the army national guard: An empirical inquiry. Suicide and Life-Threatening Behavior.

[83] Chu JP, Goldblum P, Floyd R, Bongar B (2010). The cultural theory and model of suicide. Appl Prev Psychol.

[84] Chu JP, Maruyama B, Batchelder H, Goldblum P, Bongar B, Wickham RE (2020). Cultural pathways for suicidal ideation and behaviors. Cultur Divers Ethnic Minor Psychol.

[85] Odafe MO, Talavera DC, Cheref S, Hong JH, Walker RL (2016). Suicide in racial and ethnic minority adults: A review of the last decade. Curr Psychiatry Rev.

[86] Chu JP, Robinett EN, Ma JKL, Shadish KY, Goldblum P, Bongar B. Cultural versus classic risk and protective factors for suicide. Death Stud. 2019 Jan 2;43(1):56–61. 10.1080/07481187.2018.1430085 29394156

[87] Gutierrez L, Chadwick N, Wanamaker KA (2018). Culturally relevant programming versus the status quo: A meta-analytic review of the effectiveness of treatment of Indigenous offenders. Can J Criminol Crim Justice.

[88] Marlowe DB, Shannon LM, Ray B, Turpin DP, Wheeler GA, Newell J (2018). Developing a culturally proficient intervention for young African American men in drug court: Examining feasibility and estimating an effect size for Habilitation Empowerment Accountability Therapy (HEAT). Journal for Advancing Justice.

[89] Hall GCN, Berkman ET, Zane NW, Leong FTL, Hwang W-C, Nezu AM (2021). Reducing mental health disparities by increasing the personal relevance of interventions. Am Psychol.

[90] Fowler JC (2012). Suicide risk assessment in clinical practice: Pragmatic guidelines for imperfect assessments. Psychotherapy.

[91] Bryan CJ, Rudd MD (2006). Advances in the assessment of suicide risk. J Clin Psychol.

[92] Abracen J, Gallo A, Looman J, Goodwill A (2016). Individual community-based treatment of offenders with mental illness: Relationship to recidivism. J Interpers Violence.

[93] Dewa CS, Loong D, Trujillo A, Bonato S. Evidence for the effectiveness of police-based pre-booking diversion programs in decriminalizing mental illness: A systematic literature review. PLOS ONE. 2018 Jun 19;13(6):e0199368. 10.1371/journal.pone.0199368 PMC6007921 29920560

[94] Lindquist-Grantz R, Mallow P, Dean L, Lydenberg M, Chubinski J. Diversion programs for individuals who use substances: A review of the literature. Journal of Drug Issues. 2021 Jul 1;51(3):483–503.

[95] Steadman HJ, Naples M (2005). Assessing the effectiveness of jail diversion programs for persons with serious mental illness and co-occurring substance use disorders. Behav Sci Law.

[96] Glaser W, Florio D (2004). Beyond specialist programs: A study of the needs of offenders with intellectual disability requiring psychiatric attention. J Intellect Disabil Res.

[97] Lunsky Y, Gracey C, Koegl C, Bradley E, Durbin J, Raina P. The clinical profile and service needs of psychiatric inpatients with intellectual disabilities and forensic involvement. Psychology, Crime & Law. 2011 Jan;17(1):9–23.

[98] Bailey ZD, Feldman JM, Bassett MT (2021). How structural racism works — Racist policies as a root cause of U.S. racial health inequities. N Engl J Med.

[99] Clark HW, Power AK (2005). Women, co-occurring disorders, and violence study: A case for trauma-informed care. J Subst Abuse Treat.

[100] Miller WR, Rose GS (2009). Toward a theory of motivational interviewing. Am Psychol.

[101] Watson DP, Shuman V, Kowalsky J, Golembiewski E, Brown M (2017). Housing First and harm reduction: A rapid review and document analysis of the US and Canadian open-access literature. Harm Reduct J.

[102] Asarnow JR, Goldston DB, Tunno AM, Inscoe AB, Pynoos R (2020). Suicide, self-harm, & traumatic stress exposure: A trauma-informed approach to the evaluation and management of suicide risk. Evidence-Based Practice in Child and Adolescent Mental Health.

[103] Czyz EK, King CA, Biermann BJ (2019). Motivational interviewing-enhanced safety planning for adolescents at high suicide risk: A pilot randomized controlled trial. J Clin Child Adolesc Psychol.

[104] Chapman JF, Ford JD (2008). Relationships between suicide risk, traumatic experiences, and substance use among juvenile detainees. Arch Suicide Res.

[105] Gunter TD, Chibnall JT, Antoniak SK, Philibert RA, Hollenbeck N (2011). Predictors of suicidal ideation, suicide attempts, and self-harm without lethal intent in a community corrections sample. J Crim Just.

[106] Desseilles M, Perroud N, Guillaume S, Jaussent I, Genty C, Malafosse A (2012). Is it valid to measure suicidal ideation by depression rating scales?. J Affect Disord.

[107] Xiao Y, Lindsey MA. Racial/ethnic, sex, sexual orientation, and socioeconomic disparities in suicidal trajectories and mental health treatment among adolescents transitioning to young adulthood in the USA: A population-based cohort study. Adm Policy Ment Health. 2021;48(5):742–56. 10.1007/s10488-021-01122-w PMC7904031 33629220

[108] Fisher R, O’Donnell D, Ray B, Rusyniak D (2016). Police officers can safely and effectively administer intranasal naloxone. Prehosp Emerg Care.

[109] Phalen P, Ray B, Watson DP, Huynh P, Greene MS (2018). Fentanyl related overdose in Indianapolis: Estimating trends using multilevel Bayesian models. Addict Behav.

[110] Mbaba M, Brown S-E, Wooditch A, Kiss M, Murphy A, Kumari S (2018). Prevalence, diagnosis, and treatment rates of mood disorders among opioid users under criminal justice supervision. Subst Use Misuse.

[111] Lyons JS (2009). Communimetrics: A communication theory of measurement in human service settings.

[112] Graham JW. Missing data analysis: Making it work in the real world. Annu Rev Psychol. 2009 Jan;60(1):549–76. 10.1146/annurev.psych.58.110405.085530 18652544

